# Mixed connective tissue disease: Not always an obvious diagnosis

**DOI:** 10.1002/ccr3.3045

**Published:** 2020-06-17

**Authors:** Safa Rahmouni, Kaouther Maatallah, Hanene Ferjani, Leila Abid, Dhia Kaffel, Wafa Hamdi

**Affiliations:** ^1^ Department of Rheumatology Kassab Orthopedics Institute Ksar Saïd Tunisia; ^2^ Department of Anatomic Pathology Kassab Orthopedics Institute Ksar Saïd Tunisia

**Keywords:** anti‐U1‐RNP autoantibody, capillaroscopy, mixed connective tissue disease, rheumatoid arthritis, systemic sclerosis

## Abstract

Mixed connective tissue disease (MCTD) is characterized by a mixture of clinical features. The initial presentation is often incomplete, and the features of MCTD usually develop as the disease evolves. We present a 37‐year‐old female patient with overlapping symptoms. The most plausible diagnosis was MCTD.

## Graphical abstract

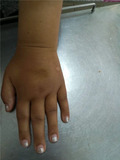

## 1 INTRODUCTION

The diagnosis of rheumatic diseases can be challenging. There is considerable overlap between the symptoms. Besides, laboratory markers are not specific and may occur in different diseases. Thus, many patients do not carry a well‐defined diagnosis.

Patients with an unclassifiable clinical picture are usually diagnosed as having “undifferentiated connective tissue disease” (UCTD).

These patients need to be followed closely in order a identify new symptoms since they might evolve into a distinct disease. Several factors may be predictive of the development of a certain disease. For instance, anti‐U1‐RNP autoantibodies are the biological signature of mixed connective tissue disease (MCTD) and may precede the clinical symptoms.^1^

While the terminology may be misleading, MCTD is now recognized as a distinct entity.^2^

We describe the case of a young female patient with overlapping symptoms highly suggestive of MCTD.

## 2 RESULTS

A 37‐year‐old lady presented with complaints of symmetrical pain of the small joints of her hands for three years with puffy hands and sicca symptoms.

There was no history of skin rashes, photosensitivity, or Raynaud syndrome.

She had no family history of similar complaints, rheumatic diseases, psoriasis, nor connective tissue diseases.

**Figure 1:**
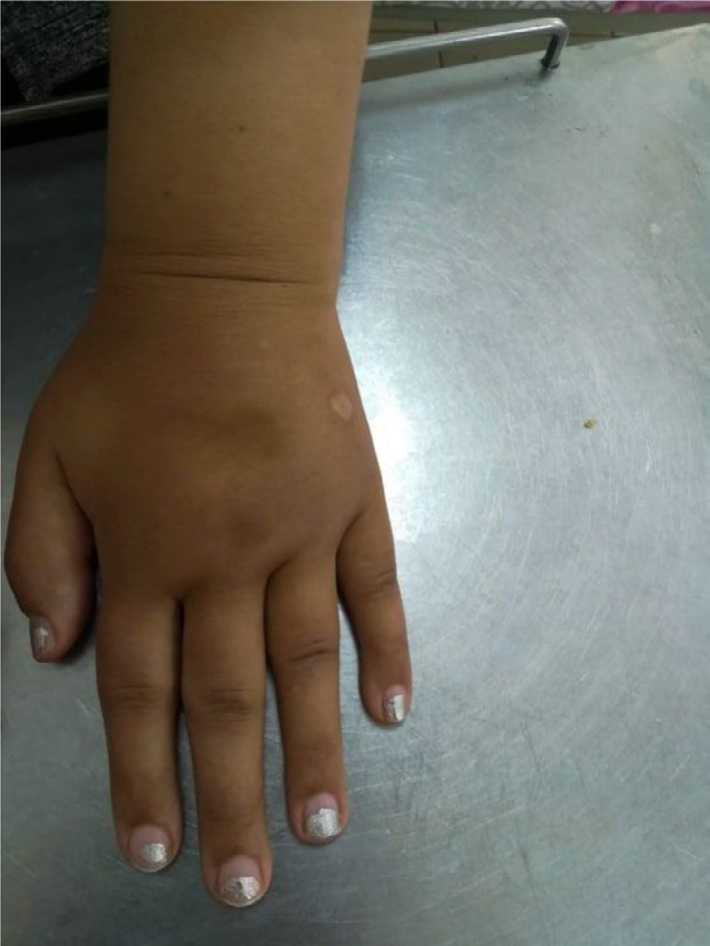
Puffy hands with pitting edema

On physical examination, she showed puffy fingers (Figure 1) with bilateral swelling and tenderness of the distal interphalangeal (DIP), proximal interphalangeal (PIP), metacarpophalangeal (MCP), and wrist joints without joint limitation or deformity.

The remaining joints did not show any inflammatory signs.

There were no skin change characteristics of a connective tissue disease, notably sclerosis.

The ophthalmologic examination found an abnormal Schirmer's test (≤ 5 mm/5 min) and superficial punctate keratitis.

Laboratory examination revealed an increased erythrocyte sedimentation rate (ESR) (68 mm/h) with normal CRP and blood cell count.

The protein electrophoresis detected the presence of polyclonal hypergammaglobulinemia (23.7g/L). On immunological examination, the antinuclear antibodies were positive (titer:1/880) with a speckled pattern along with positive anti‐SS‐B antibodies (++). Both rheumatoid factor and anticyclic citrullinated peptide antibodies (anti‐CCP) were negative.

As for the human leukocyte antigens (HLA) typing, she had HLA‐A2, B8, and B44.

**Figure 2:**
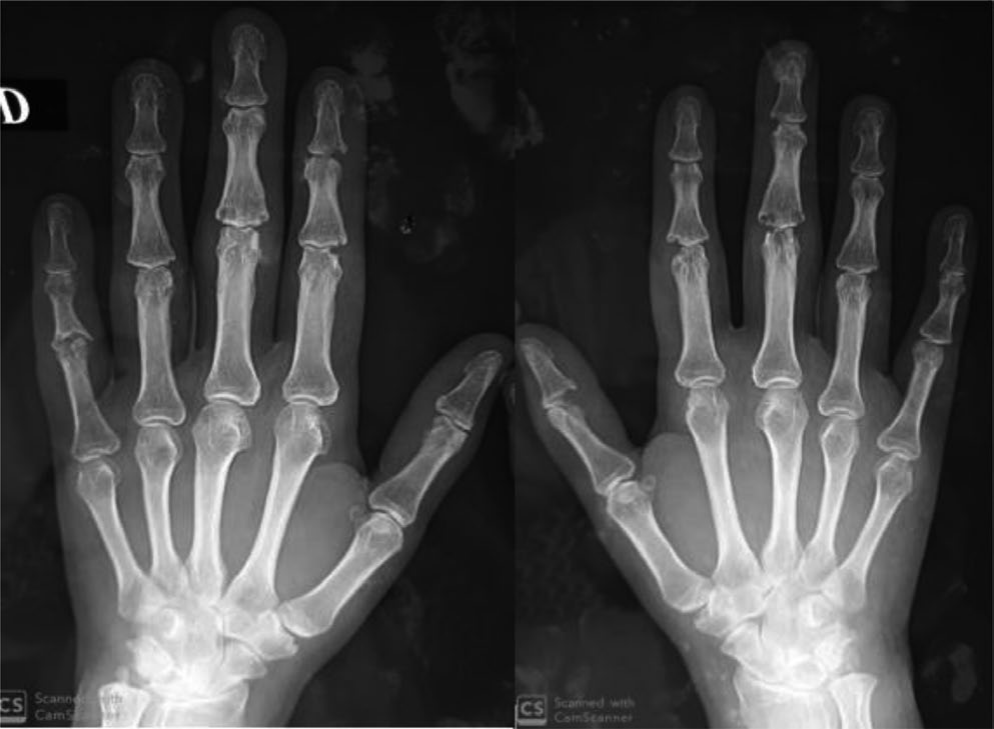
X‐rays of hands at presentation: Joint space narrowing and marginal erosions of the DIP and PIP joints. Note the soft‐tissue swelling as well

Imaging of the hands showed juxta‐articular osteopenia, erosions, and joint space narrowing in the carpal, PIP, and DIP joints (Figure 2).

We did not find any digital calcinosis nor acro‐osteolysis.

Radiographs of the feet showed erosions in the first and fifth metatarsal heads.

At this point, the diagnosis of seronegative rheumatoid arthritis (RA) was made based on clinical findings and imaging evidence of erosions, particularly of the fifth metatarsal heads.

Oral prednisolone was initiated, at the dose of 30 mg resulting in improvement of the puffy hands and arthritis.

Methotrexate was also given at the dose of 10 mg/wk.

When tapering the corticosteroids to 10 mg, the patient experienced a relapse of the joint pain and edema. Then, she was lost to follow‐up for 1 year.

After one year, she presented at our clinic with the same complaints: arthralgia involving the small joints of the* *hands and feet.

Clinical examination revealed puffy hands with arthritis of the third DIP joints bilaterally, PIP, and MCP joints. We found a painless mass on the ulnar side of the left wrist, measuring 30 × 20 mm. We also noted firm and painless nodules located next to the first metatarsal joints bilaterally.

Musculoskeletal ultrasound (US) of the hands revealed multiple erosions and synovitis (of the wrist, MCP joints, or PIP joints and DIP joints) with tenosynovitis of the common extensor tendon of both hands and an echogenic mass measuring 30mm on the ulnar side of the wrist.

Echogenic nodules were also seen next to the medial side of the first MTP joints.

**Figure 3:**
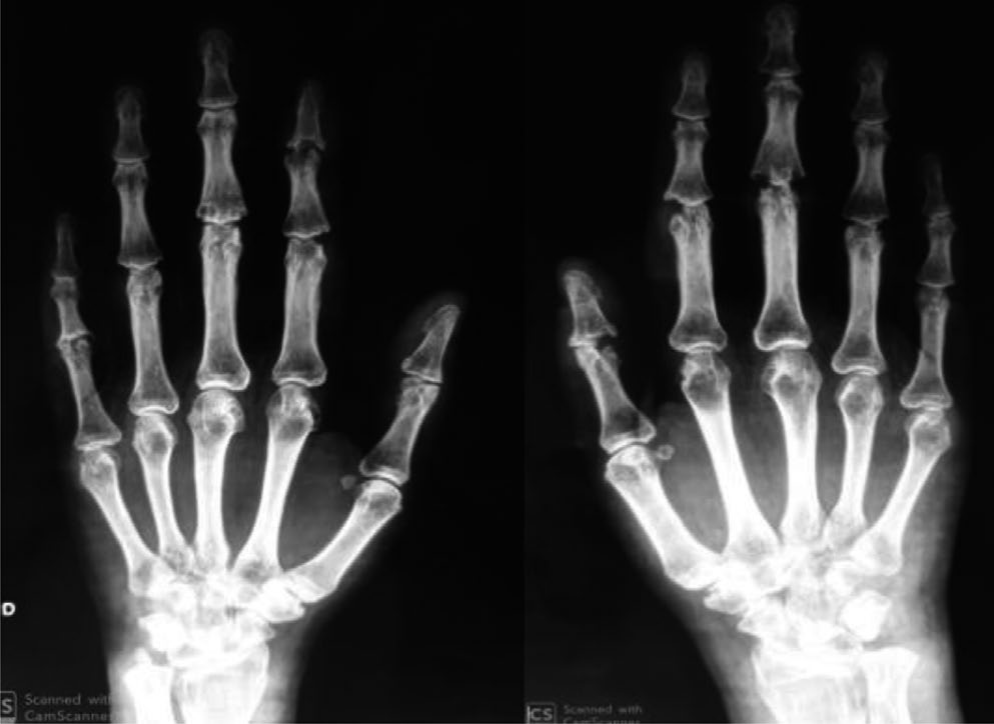
X‐ray of hands shows progression of structural damage

X‐ray of the hands and feet showed progression of the structural damage (Figure 3).

Repeated immunological studies revealed positive antinuclear antibodies (titer 1/3200) with a speckled pattern. Anti‐U1 RNP antibodies (+++), anti‐SS‐A antibodies (++++), and anti‐SS‐B antibodies (++) were also detected, without anti‐DNA, anti‐Sm, anti‐Scl‐70, and anti‐centromere antibodies. The RF and anti‐CCP antibodies remained negative.

**Figure 4:**
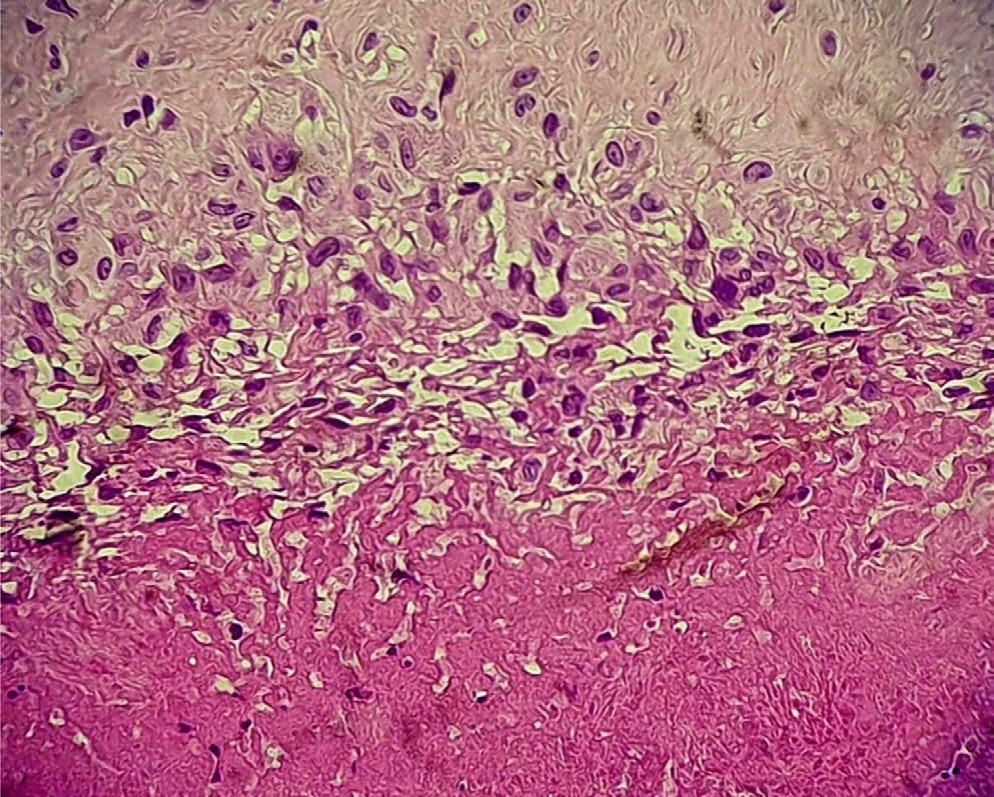
Intradermal nodule showing eosinophilic collagen surrounded by palisading epithelioid macrophages. (Hematoxylin and eosin staining, original magnification ×200)

The histological examination of the feet nodules showed eosinophilic degenerated collagen surrounded by palisading epithelioid macrophages and fibrosis tissue, findings consistent with rheumatoid nodules (Figure 4).

The minor salivary gland biopsy (MSGB) showed focal lymphocytic sialadenitis with a Chisholm*‐*Mason grade of 4 (Focus Score > 1).

The nail fold capillary microscopy showed a variety of morphological changes including an important reduction in capillaries, avascular areas, and edema.

The upper gastrointestinal endoscopy, the respiratory function test, and the echocardiography came back normal.

Although our patient did not fulfill the diagnostic criteria for MCTD, this was the most plausible diagnosis given the combined clinical and laboratory finding characteristic of rheumatoid arthritis and systemic sclerosis (SSc), mainly the abnormal capillary findings as well as the positive anti‐U1 RNP antibodies.

Sjögren's syndrome was also diagnosed by the 2016 ACR‐EULAR criteria.

The patient was given oral prednisolone at the dose of 10 mg and methotrexate at a dose of 20 mg/wk.

After one year of follow‐up, the patient was symptom‐free and did not develop any additional symptoms.

## 3 DISCUSSION

In the present study, we have described a case of a young female patient who presented initially with severe erosive arthritis with DIP involvement, puffy hands, and sicca syndrome. During the course of the disease, she developed rheumatoid nodules on the medial side of the left wrist and next to the medial aspect of the first MTP joints bilaterally, with the occurrence of anti‐U1RNP antibodies.

Despite the involvement of the DIP joints, the initial presentation was suggestive of RA.

However seldom, DIP involvement has been sparsely reported in severe and long‐standing RA.^3^ In the previous studies, DIP involvement was reported in 15% of RA patients.^4^, ^5^

However, unlike our case, it usually occurs in older patients (mean age at 50 years old) with longer disease duration (10 years).^4^

During the course of the disease, other features appeared.

Anti‐U1 RNP antibodies appeared one year after the initial presentation. These antibodies are the hallmark of MCTD, and their emergence often precedes the onset of the clinical disease. A high titer of anti‐RNP antibodies in any patient with features of UCTD is highly predictive of evolution into MCTD.^6^ In a prospective study on patients with anti‐U1‐RNP antibodies, 60% of patients presented with symptoms compatible with a specific connective tissue disease other than MCTD. After a mean follow‐up of 6 years, 90% fulfilled the criteria for MCTD.^7^

At this point, the presentation was highly suggestive of MCTD.

The clinical features of MCTD often develop over several years, and the complete clinical findings are rarely present at the start of the disease. The most common manifestations of MCTD at onset are Raynaud's phenomenon, polyarthritis, and swollen fingers or hands.^8^ This was partially in line with our observation.

Regarding joint involvement in MCTD, it is a common feature, affecting up to 70% of patients. Arthritis associated with MCTD has been described as mild, nonerosive, and nondeforming.^9^, ^10^ However, a broader spectrum of joint disease may also occur, including deforming arthritis,^11^ mutilans‐type erosive arthritis,^12^ and RA‐like arthritis. The DIP involvement as described in our patient has been previously reported as well.^13^, ^14^

In patients with MCTD, RF is a risk factor for erosive joint disease.^11^, ^15^ However, as in RA, erosions may occur in the absence of RF and anti‐CCP antibodies.

During the course of the disease, our patient presented with nodules suggestive of rheumatoid nodules. While the diagnosis of typical rheumatoid nodules does not require histological proof, a skin biopsy was conducted given the unusual location. Rheumatoid nodules are highly specific of RA, but they are not pathognomonic and may occur in other rheumatic diseases, notably MCTD.^16^

With the diagnosis of MCTD in mind, we conducted nail fold capillary microscopy which revealed typical SSc‐like findings. In fact, early microvascular abnormalities may appear before the development of Raynaud's phenomenon.

The abnormalities we found (avascular areas with an overall reduced capillary density) are reported to be predictors of the transition to a connective tissue disease.^17^

It should be kept in mind that sclerodermatous involvement usually occurs later in the disease course of MCTD.^18^

Furthermore, our patient presented with sicca symptoms and had anti‐SSA and anti‐SSB antibodies. Sicca symptoms can also be a feature of MCTD, affecting nearly 40% of patients.^19^ Additionally, anti‐SSA and anti‐SSB may be present in MCTD.^20^

An authentic Sjogren syndrome is not rare in MCTD,^21^ though some authors claimed that it may be underdiagnosed as a condition associated with MCTD.

Our patient fulfilled the 2016 ACR‐EULAR criteria for SS (Schirmer's test ≤5 mm/5 min, positive anti‐SSA antibodies, and positive biopsy results).

We also conducted a careful work‐up to identify potential organ manifestations suggestive of connective tissue disease, but it came out negative.

For a long time, MCTD was considered a transient phase of a connective tissue disease, not yet having reached its final expression. However, several genetic, serological, and clinical features argue for MCTD as a distinct rheumatic disease and against the concept of an early form of other connective tissue diseases.^2^, ^22^ MCTD is associated with HLA‐DR1, HLA‐DR4, and to a lesser degree with HLA‐DR2.^23^, ^24^ Similar to MCTD, RA is associated with HLA‐DR1 and HLA‐DR4. Apart from erosive arthritis, RA usually does not show autoantibodies against U1‐RNP.

There is no one widely accepted set of classification criteria; several criterion sets have been tested successfully, including Sharp's criteria, the Kasukawa diagnostic criteria, and the Alarcón‐Segovia criteria.^25^, ^26^, ^27^ Diagnostic criteria may help define patients with MCTD, but in several cases, patients may not fulfill diagnostic criteria at their initial presentation.

Mixed connective tissue disease evolves over time, and patients typically develop new clinical and laboratory features in the course of the disease. Thus, patients might display a few features of the disease and may not fulfill the classification criteria for MCTD at their initial presentation, as noted in this observation.

In some cases, the diagnosis of MCTD can be made based on expert opinion.^18^

Regarding our observation, the patient did not report Raynaud syndrome, a major feature of MCTD. However, knowing that early microvascular abnormalities may appear before the development of Raynaud's phenomenon, we suggest that the nail fold findings could be used instead of Raynaud phenomena. In this case, the patient would fulfill Alarcon‐Segovia's as well as the Kahn criteria: anti‐RNP antibodies, synovitis, swollen hands, and abnormal capillaroscopy with scleroderma pattern.

The management of our patient relied on interpolation of treatment guidelines for other connective tissue diseases.^28^

Musculoskeletal manifestations were the main complaints of our patient, and she had severe erosive arthritis.

The arthritis of CTD usually responds to steroids, but in severe destructive cases, other drugs such as methotrexate or leflunomide may be prescribed.

In summary, the concept of MCTD is controversial because the clinical entity is still obscure.

Diagnostic criteria may help define patients with MCTD. However, in several cases, patients do not satisfy any criteria.

Our patient presented typical RA‐like symptoms (erosive arthritis, rheumatoid nodules) and typical SSc‐like findings (abnormal nail fold capillary and puffy fingers) along with anti‐U1RNP antibodies. She also had associated Sjogren syndrome.

Given that clinical features of MCTD tend to occur sequentially over several years, we highlight the importance of a long‐term follow‐up to better define the natural course of the disease and the potential evolution toward other connective tissue diseases.

Another key message is to consider the diagnosis of MCTD in patients presenting as RA with unusual features since arthritis in MCTD may be both erosive and deforming.

## CONFLICT OF INTEREST

None declared.

## AUTHOR CONTRIBUTIONS

SR: drafted the manuscript with the help of KM. HF: was responsible for the analysis and interpretation of data. LA: was responsible for the pathology report which was of great help for the diagnosis. DK: revised the manuscript. WH: approved the final manuscript.
